# Transcriptome sequencing of facial adipose tissue reveals alterations in mRNAs of hemifacial microsomia

**DOI:** 10.3389/fped.2023.1099841

**Published:** 2023-02-13

**Authors:** Bingyang Liu, Wei Liu, Shanbaga Zhao, Lunkun Ma, Tianying Zang, Changjin Huang, Kaiyi Shu, Hengbin Gao, Xiaojun Tang

**Affiliations:** ^1^ Department of Craniomaxillofacial Surgery, Plastic Surgery Hospital, Chinese Academy of Medical Sciences and Peking Union Medical College, Beijing, China; ^2^ State Key Laboratory of Crop Biology, College of Life Sciences, Shandong Agricultural University, Tai’an, China

**Keywords:** hemifacial microsomia, HAND2, HOXB2, facial adipose tissue, RNA sequencing

## Abstract

Hemifacial microsomia (HFM) is a common congenital malformation of the craniofacial region, including mandibular hypoplasia, microtia, facial palsy and soft tissue deficiencies. However, it remains unclear which specific genes are involved in the pathogenesis of HFM. By identifying differentially expressed genes (DEGs) in deficient facial adipose tissue from HFM patients, we hope to provide a new insight into disease mechanisms from the transcriptome perspective. RNA sequencing (RNA-Seq) was performed with 10 facial adipose tissues from patients of HFM and healthy controls. Differentially expressed genes in HFM were validated by quantitative real-time PCR (qPCR). Functional annotations of the DEGs were analyzed with DESeq2 R package (1.20.0). A total of 1,244 genes were identified as DEGs between HFM patients and matched controls. Bioinformatic analysis predicted that the increased expression of *HOXB2* and *HAND2* were associated with facial deformity of HFM. Knockdown and overexpression of *HOXB2* were achieved with lentiviral vectors. Cell proliferation, migration, and invasion assay was performed with adipose-derived stem cells (ADSC) to confirm the phenotype of *HOXB2*. We also found that PI3K−Akt signaling pathway and human papillomavirus infection were activated in HFM. In conclusion, we discovered potential genes, pathways and networks in HFM facial adipose tissue, which contributes to a better understanding of the pathogenesis of HFM.

## Introduction

Hemifacial microsomia (HFM) is one of the most common congenital craniofacial defects, incidence of which ranges from 1:13,500 to 1:5,600 (1), second only to cleft lip and palate. HFM, which was first described in 1,881 (OMIM *164210), typically affects the external ear, middle ear, mandible and temporomandibular joint, mastication and facial muscles, and other facial soft tissues on the affected side. In some affected patients, anomalies may also include in cardiac, vertebral, and central nervous system in addition to craniofacial anomalies. They significantly affect facial appearance and physiological function, posing a substantial psychological and financial burden on families.

The precise etiology and physiopathology of HFM are far from being completely understood and both environmental and genetic factors can be considered to interpret the symptoms of HFM. In terms of environmental factors, the embryo is vulnerable to teratogens, such as thalidomide (2), retinoic acid (3), vasoactive medications, and alcohol (4), which may result in permanent congenital malformations, including HFM. In addition, maternal diabetes (5), multiple gestations, and vaginal bleeding during pregnancy (4) are also major risk factors for HFM. Despite the fact that most cases of HFM are sporadic, familial occurrence suggests a genetic predisposition. Most cases of familial occurrence show an autosomal dominant transmission, which accounts for 2% to 10% of cases (6). Currently, most studies on HFM are limited to evaluating one or more genes for expression alterations, but systematic research on differentially expressed genes or primary pathways is lacking. As a result, HFM is an enigmatic condition with an unknown etiology and poorly understood pathogenesis. It is essential to understand how disease develops and pathogenesis occurs by interpreting transcriptomes. Reports have not been found about the global transcriptome abnormalities of the facial adipose tissue from patients with HFM. Next-generation sequencing (NGS) is well-established for deciphering the transcriptome using high-throughput and quantitative methods (7). The aim of our study is to identify differentially expressed genes (DEGs) and molecular pathways in facial adipose tissue from patients with HFM, which will provide new insights into disease mechanisms at the molecular level.

## Materials and methods

### Patients and clinical samples

Patients were diagnosed and recruited based on typical clinical symptoms of mandibular, ear and facial soft tissue hypoplasia, physical examination results, and imaging reports including x-ray and CT reports. All samples were collected from Department of maxillofacial surgery, Plastic Surgery Hospital, Chinese Academy of Medical Sciences, Peking Union Medical College. All participants enrolled in our present study were Han Chinese, Asia population.

Six patients with HFM and four healthy controls were enrolled in our study. All patients' adipose tissue samples in our manuscript were collected through scar excision when removing the mandibular distractor surgery. The adipose tissue from controls was also collected through the buccal fat pad resection procedure from patients with facial fat accumulation. After taking adipose tissue away from operating table, we dissected it into small pieces with surgical scissors. Samples were maintained in centrifuge tubes with RNAlater (Ambion Inc) and stored at −80°C as the first part, preserved in cryotub.

According to the Declaration of Helsinki Principles, the protocols for the study were approved by the Ethics Review Committee of Plastic Surgery Hospital. Written informed consent was obtained from participants.

### RNA sequencing (RNA-seq)

According to the manufacturer's instructions, total RNA was isolated from adipose tissues using the RNeasy Lipid Tissue Mini Kit (QIAGEN). In accordance with the manufacturer's instructions, stranded total RNA LT sample prep kit (Illumina) was used to prepare the compatible library. A Bioanalyzer (Agilent) was used to analyze the quality and concentration of all libraries. Sequencing of mRNA was performed on an Illumina Hiseq 2,500 sequencing system (Illumina), and 150-bp paired-end FASTQ files were generated.

### cDNA preparation and quantitative PCR

Total RNA was isolated with the TRIzol reagent (Thermo Fisher Scientific) according to the manufacturer's protocol. The RNA concentration and purity were evaluated with NanoDrop ND-2000 spectrophotometer (Thermo Fisher Scientific Inc). With 1 µg of RNA in the 20 µl reaction system, reverse transcription reactions were performed with PrimeScript Reverse Transcriptase (Takara Bio) according to the manufacturer's instructions.

To validate the confidence of RNA-Seq, several differentially expressed genes were selected and analysed by quantitative real-time PCR (qPCR) utilizing the SYBR Premix EX Taq reagent (Takara) in a QuantStudio 7 Flex Real-Time PCR System (Applied Biosystems). Primers (Supplementary Table S1) were designed for the coding sequences of the candidate genes in Primer 3 software (http://frodo.wi.mit.edu/cgibin/primer3). GAPDH was used as the internal control. qPCR replicates were performed in a final volume of 10 µl containing primers, SYBR Premix EX Taq reagent (Takara) and cDNA templates. All quantitative PCRs were performed for three biological replicates. The relative expression levels of the candidate genes were calculated as the averaged normalized Ct value of each sample compared with the GAPDH Ct value of the corresponding sample based on the 2−ΔΔCt method.

### Detection of differentially expressed genes

Our RNA expression levels were estimated using HISAT2 (version 2.1.0). Genome sequence data (hg19, Genome Reference Consortium GRCh37) and annotation data were obtained from UCSC website (https://genome.uscs.edu). With featureCounts (v1.5.0-p3), we calculated transcript counts at the gene level and relative abundances in FPKM (fragments per kilobase of exon per million fragments mapped). We build Principal Component Analysis (PCA) and identified 1,234 differential expressed genes (DEGs) by using DEseq2 (*P-*value <0.05, log2Foldchange >2) (8). Heatmap show the gene expression pattern in different groups, all DEGs expression level were scale to −2 and 2 based on Z-score calculation. Diagrams were visualized by ggplot2 in R version 4.1.0.

### Gene annotation: gene ontology and pathway analysis

The KEGG (Kyoto Encyclopedia of Genes and Genomes database) and gene ontology (GO) pathway enrichment analysis was performed using ClusterProfiler package from BioConductor (http://www.bioconductor.org/). A hypergeometric exact test was used to classify enrichment GO categories and KEGG pathways. Only terms with a *P*-value 0.05 were considered enriched. According to the rank of *P-*value, we display the top 20 GO enriched terms for both upregulated and downregulated genes.

### Cell lines, transfection and construction of lentiviral vectors

The buccal fat pad from healthy controls were collected in D-Hanks solution with 1,000 U/L penicillin streptomycin (Solarbio, Beijing, China). An equal volume of 0.25% of EDTA trypsin supplemented with 0.1% of type I collagenase solution was added, and the samples were gently vibrated in a 37°C incubator shaker for 45 min. Cells were collected and centrifuged at 1,000 rpm for 10 min; the supernatant was removed, and the cells were resuspended with DMEM supplemented with 10% FBS. After ﬁltering using a 100-mesh sieve, cells were transferred into a culture dish and incubated in 37°C with 5% CO 2 for 24 h. Cells were observed using a phase-contrast microscope daily. Cell morphology and proliferating rates were recorded, and when cells reached 90%–95% conﬂuence, cells were digested with 0.25% EDTA trypsin and subcultured into several culture dishes. Lentiviral interference vectors, pCMV.GFP&PR.U6 (sh-, interference vector), and lentiviral overexpression vector, VP001-CMV-MSC-3flag-EF1-ZsGreen-T2A-PURO (oe-, overexpression vector), were purchased from Sangon Biotech (Shanghai, China) and General Biosystems (Anhui,China). A lentiviral-based *HOXB2* interference vector, a *HOXB2* overexpression vector were constructed. Lentiviral infections were performed using protocols available online (www.broadinstitute.org). Next, cells were inoculated into a six-well plate at a density of 5 × 104 cells/ml and infected with NC, si-HOXB2, vector, HOXB2-OE, respectively.

### Cell proliferation, transwell assays

For the cell proliferation test, a CCK8 Kit (Solarbio, China) was used. In 96-well plates, cells were seeded in 96-well flat-bottomed plates with each well containing 2,000 cells in 200 µl of culture medium and cultured in ambient environment described above. CCK8 reagent was added into wells, after incubation at 37°C for 24 h in a humidified incubator with 5% CO2, the proliferative ability of the cells was measured at 450 nm. For transwell migration assay, different cells were suspended in 200 µl of DMEM without FBS and seeded on the top chamber of 24-well plate-sized Transwell inserts (Corning Falcon). The lower chambers contained DMEM with 20% FBS. After incubation for 8 h, the inserts were fixed and stained with crystal violet. Cells in the upper chamber were removed with cotton swabs. The average confluence of migrated cells was analyzed by ImageJ according to three random fields captured by 100× microscope. Each experiment was conducted in triplicate.

## Result

### Clinical characteristics in the HFM and healthy control groups

The 6 patients with HFM enrolled in our study who were aged from 7 to 26. All patients were classified with Pruzansky II and III mandibular hypoplasia, including facial soft tissue deficiencies, shortened mandibular ramus, small glenoid fossa, malformed condyle, preauricular tags, microtia, excluding other concurrent clinical symptoms (epibulbar dermoids and vertebral anomalies). The 4 matched controls were all adults who aged from 20 to 25. The adipose tissue was collected when matched controls underwent buccal fat pad resection. The clinical details of the participants are provided in Supplementary Table S2.

### RNA-seq analysis and identification of differentially expressed genes

**Figure 1:**
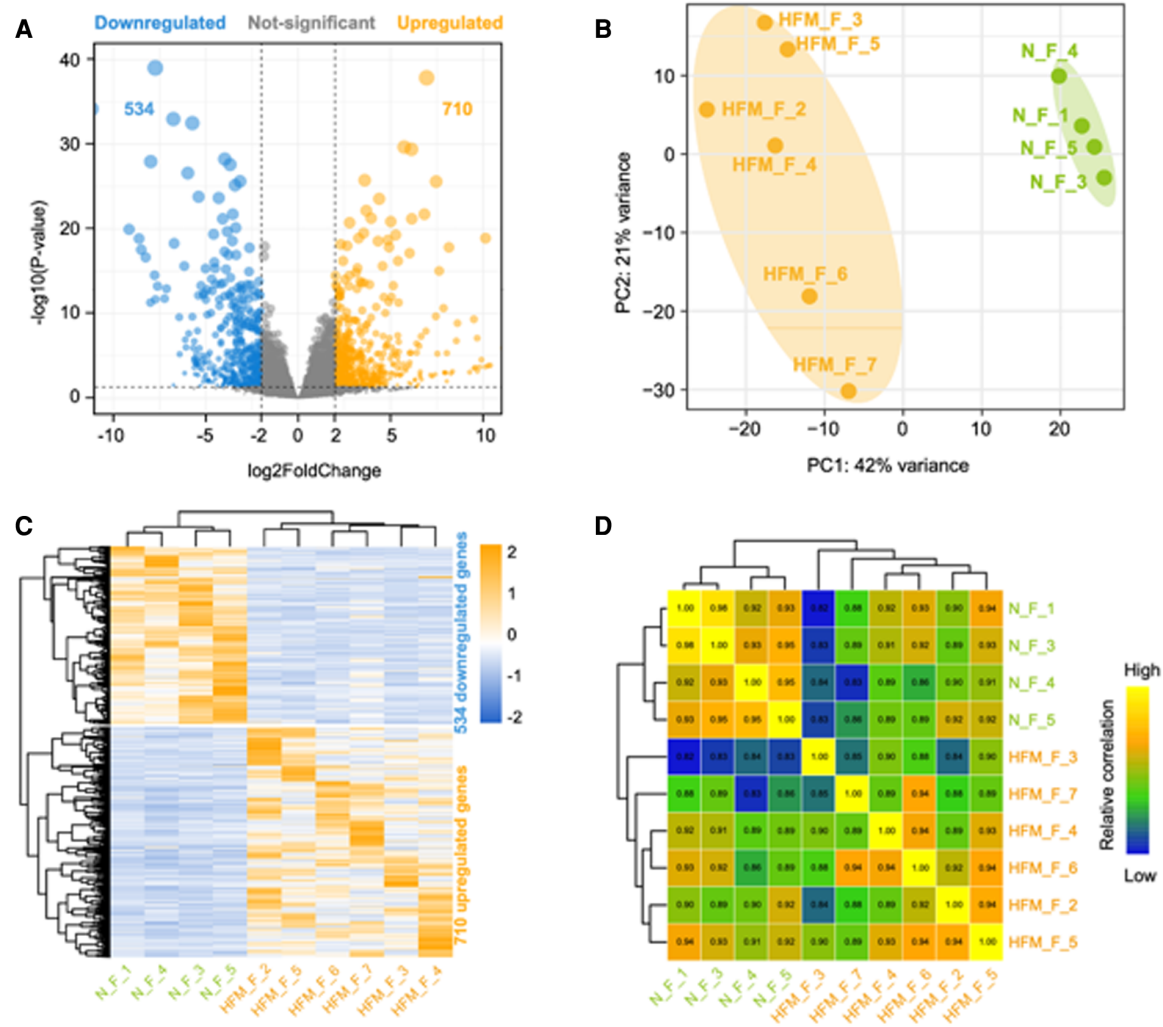
Differentially expressed genes between facial adipose tissue from paitients with HFM and matched controls identified by RNA-Seq. (**A**) Volcano plots of genes with differential expression. The x-axis represents the log 2 (fold change), and the y axis represents −log 10 (*P*-value) calculated by student's *t*-test. The orange points represent the upregulated genes and blue ones represent the downregulated genes. (**B**) Principal component analysis (PCA). (**C**) Hierarchical clustering analysis of genes with differential expression. (**D**) Heatmap summarizing correlation between control and experiment group in log2 gene expression profiles.

A transcriptomic analysis of adipose tissue from six patients with HFM and four healthy control was conducted to better understand the pathogenesis of the disease. A total of 28 million read pairs were obtained from each sample and were compared between the HFM and control groups in adipose tissue. The total number of annotated mRNAs identified was 333,341 and 1,244 mRNAs were significantly deregulated (q-value <.05; absolute value Log2 Ratio ≥1) (Figure 1A). A comparison of the adipose tissues from patients with HFM and those from controls demonstrated that 710 genes were up-regulated, and 534 genes were down-regulated. The differentially expressed mRNA data sets were analyzed using principal component analysis and hierarchical clustering. HFM adipose tissue exhibited distinct gene expression profiles compared with control adipose tissue based on PCA (Figure 1B). According to a hierarchical clustering analysis of the differentially expressed genes in adipose tissue from patients with HFM and control subjects, each gene expression pattern clustered separately (Figure 1C). Subsequently, we draw a correlation heatmap between various gene expression (Figure 1D). The results indicated that HFM has a profound impact on the expression of mRNA in the facial adipose tissue.

### Validation of mRNA expression

**Figure 2:**
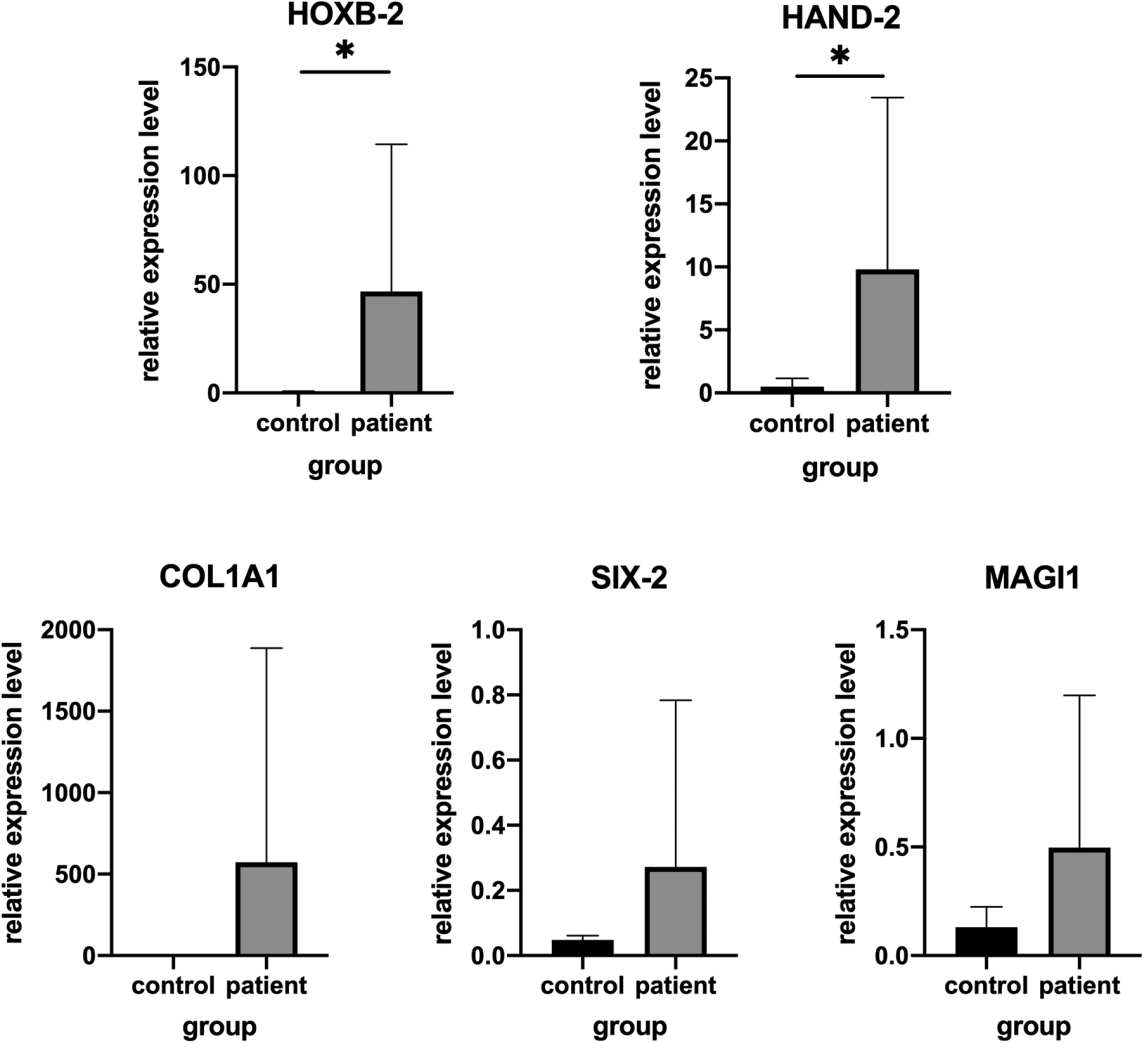
Validation of genes expression by real-time PCR in other patients and matched controls. (**A**) *HAND2*, (**B**) *HOXB2*, (**C**) *COL1A1*, (**D**) *MAGI1* and (**E**) *SIX-2*.*P < .05.

To confirm the accuracy of RNA-Seq, five genes including up-regulated (*HOXB2*, *HAND2*, *COL1A1*, *MACH1*) and down-regulated (*SIX2*) between adipose tissue from patient with HFM and controls were selected for further validation in additional set of clinical samples. Consistent with our expectation, the validation results of *HOXB2* and *HAND2* were consistent with the RNASeq data. While no significant difference (*P* > 0.05) was detected in the levels of *COL1A1*, *MACH1*, *SIX2* mRNA, according to the results of quantitative real-time PCR (Figure 2).

### Functional analysis of differentially expressed genes

Gene ontology and pathway analysis of genes were performed to find possible biological alterations related to HFM. A wide range of biological functions and signaling pathways are involved with DEGs. The immune system and the skeletal system exhibited the most significant differences, out of many involved pathways.

**Figure 3:**
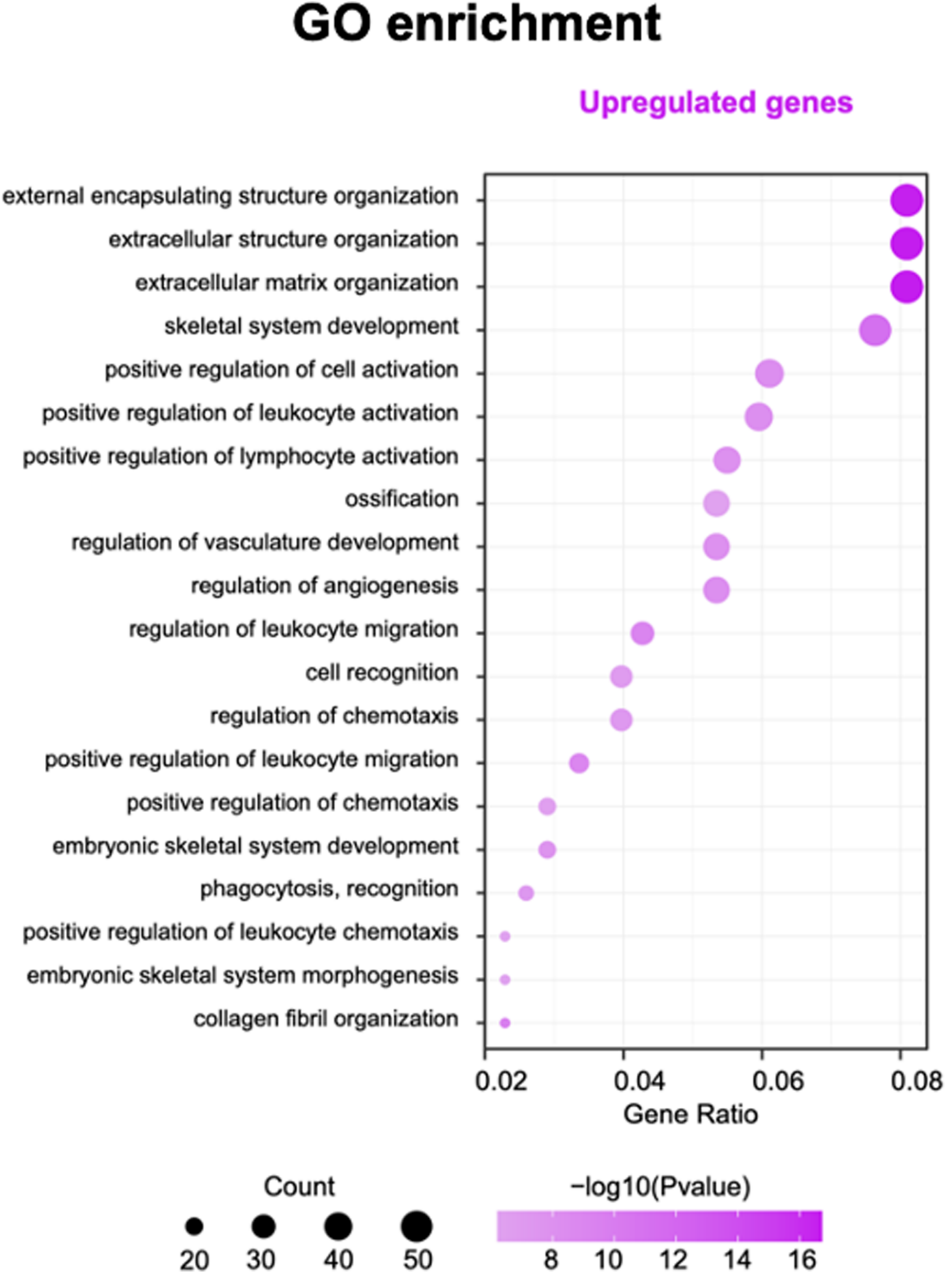
The top 10 enrichment GO (gene ontology) pathway analysis.

Based on the enrichment score, we selected the top ten items from GO (Figure. 2). the most significant biological processes related to craniofacial morphogenesis included skeletal system development, ossification, regulation of vasculature development, embryonic skeletal system development and embryonic skeletal system morphogenesis. According to KEGG analysis (Figure. 3), signal pathways including PI3K−Akt signaling pathway and Human papillomavirus infection showed significant differences. The PI3K−Akt signaling pathway was previously shown to be associated with NCCs development (9). The relationship between human papillomavirus infection and HFM has not been reported yet. Additional studies are required to further investigate these possibilities.

### Effect of *HOXB2* on ADSC

**Figure 4:**
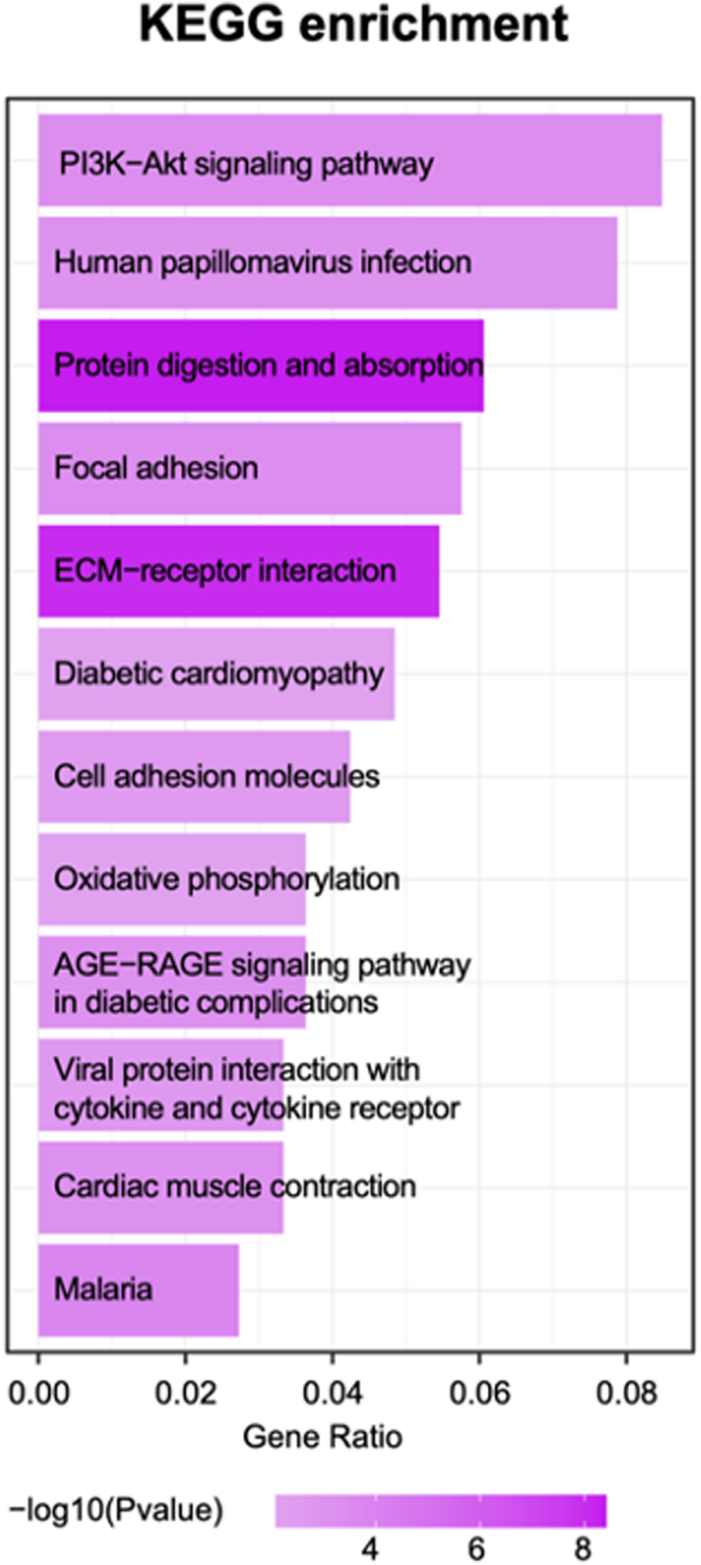
The top 10 enrichment KEGG pathway terms.

To examine the effects of *HOXB2* on ADSC proliferation and metastasis, we conducted a series of cell function experiments. *HOXB2* overexpression significantly decreased the migration and invasion of ADSC (Figures 4A–D), as well as reduced their proliferation (Supplementary Additional file S1, Figure S2B). On the contrary, Loss of HAND2 further enhanced cell migration and invasion of these ADSC (Supplementary Additional file S1, Figure S2F, S3B–D).

**Figure 5:**
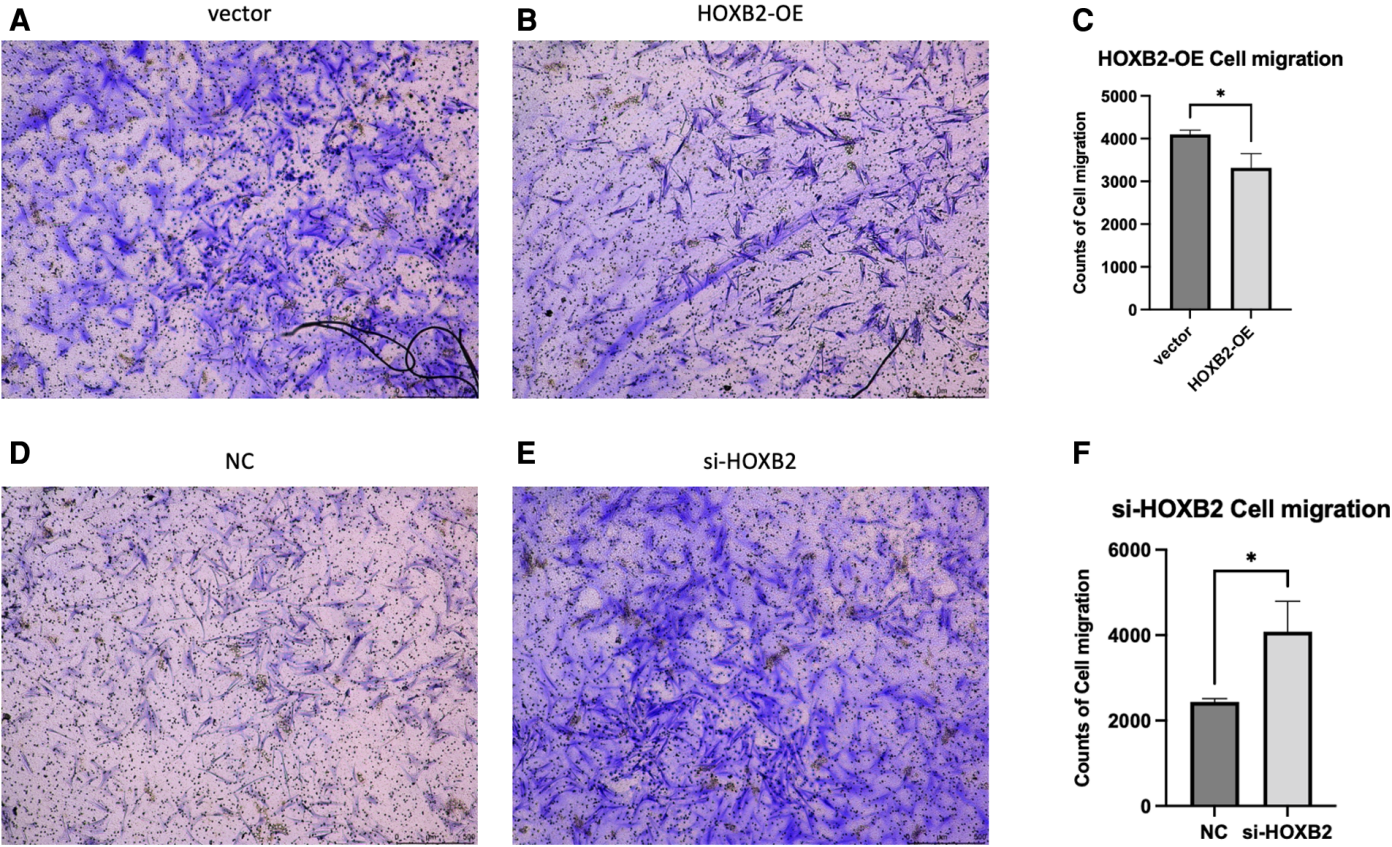
(**A–C**) overexpression of *HOXB2* inhibited ADSC migration. (**D–F**) Knockdown of *HOXB2* expression have enhanced ADSC migratory ability.

**Figure 6:**
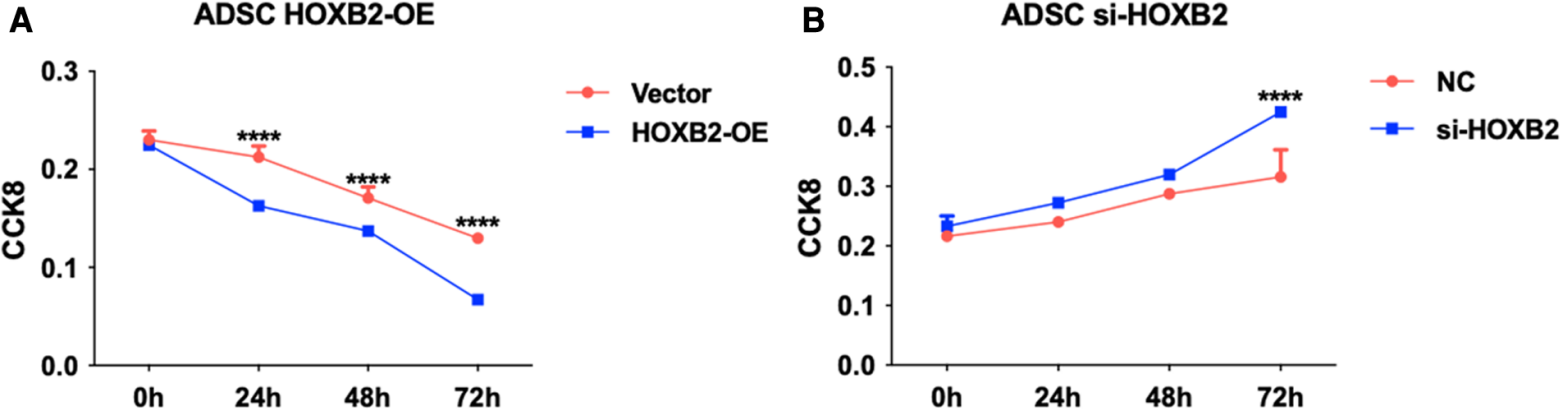
(**A**) *HOXB2* overexpression inhibited ADSC proliferation. (**B**) *HOXB2* knockdown increased the ADSC proliferation ability.

## Discussion

The molecular mechanism underlying HFM remains unclear. Numerous studies revealed that HFM has multiple contributors, including genetic, maternal, and environmental factors (10). Three main hypotheses have been suggested to explain the pathogenesis of HFM: stapedial artery abnormalities, abnormal development of cranial neural crest cells (CNCCs), and injury of Meckel's cartilage. In this study, RNA-Seq has been used for the first time to analyze the transcriptome of affected facial adipose tissue of HFM in Han Chinese.

CNCCs are highly capable of proliferating and diffusing during the embryonic period, which plays a vital role in the growth and development of craniofacial cartilage, bone tissue, as well as smooth muscle, sensory nerve, and adipose tissues (11). One-third of all congenital anomalies are related to improper development of neural crest cells (NCCs) in humans (12). The majority of abnormal craniofacial tissue involved in HFM is derivatives of CNCCs. Different parts of CNCCs have specific migration paths, forming the first and second branchial arches. Thus, it is a possible mechanism of HFM that affects the proliferation, migration, and differentiation of CNCCs (10).

To our knowledge, this is the first study using Next-generation sequencing to compare the differential expression of genomic profiles between HFM patients' and healthy controls' facial adipose tissue. Our results indicated that overexpressed of *HAND2* and *HOXB2* may resulted in the alteration of hemifacial hypoplasia in HFM patients.

*HAND2* (Heart And Neural Crest Derivatives Expressed 2) is a protein coding gene which plays an important role in limb and branchial arch development (13). *HAND2* expression in the ventral region of the branchial arch is independent of Edn1/Ednra-mediated signals (14). The expression of *HAND2* in wild type embryos is restricted to the distal mandibular mesenchyme, which is downstream of Bmp4 (15, 16). Studies indicated that overdosed BMP signaling inhibits facial skeletal formation by causing dramatic apoptosis in NCC cells (17).

According to research, high *HAND2* expression in NCCs can result in the transformation of the upper jaw into a lower jaw, resulting in the absence of a secondary palate (18). Funato et al. experiments have indicated *HAND2*-overexpressed mice showed fragmented temporal bones and malformed middle ear (malleus, incus, gonial bone, and tympanic ring) (18). *HAND2* overexpression caused hypoplastic bone formation in the cranial region, consistent with the observation that *HAND2* negatively affects mandibular ossification through direct inhibition of *RUNX2*, a master transcription factor of osteoblast-specific genes (19). These experimental results are consistent with the observed clinical manifestations in HFM patients including underdevelopment of the mandible, maxilla, ear, orbit, facial soft tissue.

*HOX* genes, which encode for homeodomain-containing transcription factors, are major inhibitors involved in patterning of animal embryos and craniofacial program carried by CNCC (20, 21). *HOXA2*, as other *HOX2* paralogs, may cooperate with *HOXB2* in the second pharyngeal arch (22). A prevalent role of *HOXA2* as its inactivation in mouse induced a mirror-image duplication of the lower jaw with two Meckel's cartilage (23, 24). Studies have also shown that patients with mutations in *HOXA2* display severe microtia, middle ear deformities and hearing loss (25, 26). In Animal experiments, the hearing impairment were also observed in *HOXB2* mutants (27). On the other HAND, *HOXA2* homozygous mutant embryos contained additional, more extensive ossification centers (23). Histological observations of *HOXB2* mutant mice detected a cartilage rod carrying a proximal protuberance, which could be interpreted as a triplicated malleus (28). Apart from triplicated malleus, the *HOXA2* mutants duplicate all skeletal elements normally derived from the first arch NCC, including ectopic Meckel's cartilage, as well as ectopic incus, malleus, tympanic, and squamous bones (24). In our experiments, it can be observed that high expression of *HOXB2* can significantly reduce ADSC migration and proliferation. Unfortunately, no animal experiments about overexpression of *HOXB2* can be found in the literature. Further verification experiments will be conducted in order to confirm the gene function. We could only speculate the overexpression of *HOXB2* were possibly in accordance with the clinical symptoms in HFM.

For the first time, we identified two mRNAs that may participate in the pathogenesis of HFM, providing a new avenue for future research. Suppressing the expression of genes, such as *HOXB2* and *HAND2*, might be a promising therapeutic approach to HFM. However, there are several limitations in our study. First of all, the sample size is small, and more samples will be collected for further study. In addition, we have not provided experimental evidence to support the role of genes. Furthermore, as the samples were collected with a wide range of ages, a comparison of HFM samples to normal adipose tissue may reveal different genes due to the tissues' developmental stages but not because of differences between them. In summary, it is necessary to further explore the underlying roles of the identified pathways and molecules in HFM.

## Conclusion

As a result, we identified 1,275 DEGs in adipose tissue of HFM patients along with key pathways and networks. It is the first study to report that HFM is related to high expression of *HAND2*and *HOXB2*, providing an important basis for further mechanistic investigations of HFM. A detailed study of these two genes in HFM pathogenesis will be conducted in the near future.

## Data Availability

The datasets presented in this study can be found in online repositories. The names of the repository/repositories and accession number(s) can be found below: https://ngdc.cncb.ac.cn/gsa-human/s/QUhld0aT, HRA002742.
