# Supplementary material for: Transcriptome sequencing of facial adipose tissue reveals alterations in mRNAs of hemifacial microsomia
Source: Front Pediatr. 2023 Feb 13;11:1099841. doi: 10.3389/fped.2023.1099841 (PMC9968928; doi:10.3389/fped.2023.1099841)
Supplement: Supplementary file 1 [file Table1.docx]

Supplementary Material

# Supplementary Tables

**Table S1 The detailed clinical information of participants**

| **Serial Number** | **Gender** | **Age** | **Pruzansky Classification** | **Experiments** |
| --- | --- | --- | --- | --- |
| HFM2 | Male | 10 | Ⅱa | RNA-Seq |
| HFM3 | Male | 9 | Ⅲ | RNA-Seq |
| HFM4 | Female | 10 | Ⅱb | RNA-Seq |
| HFM5 | Female | 26 | Ⅱa | RNA-Seq |
| HFM6 | Female | 8 | Ⅱb | RNA-Seq |
| HFM7 | Male | 11 | Ⅱb | RNA-Seq |
| HFM1 | Female | 10 | Ⅱa | qRT-PCR |
| HFM8 | Female | 10 | Ⅱb | qRT-PCR |
| HFM9 | Male | 9 | Ⅲ | qRT-PCR |
| HFM10 | Male | 6 | Ⅱa | qRT-PCR |
| HFM11 | Female | 10 | Ⅱb | qRT-PCR |
| HFM12 | Female | 7 | Ⅱa | qRT-PCR |
| HFM13 | Male | 7 | Ⅱa | qRT-PCR |

**Table S2 Sequences of PCR primers used in this study**

| **Genes** | **Sequences (5'-3')** |
| --- | --- |
| G1-HOXB-2_F | CGCCAGGATTCACCTTTCCTT |
| G1-HOXB-2_R | CCCTGTAGGCTAGGGGAGAG |
| G2-HAND-2_F | CGCCGACACCAAACTCTCC |
| G2-HAND-2_R | TCGCCATTCTGGTCGTCCT |
| G3-COL1A1_F2 | GAGGGCCAAGACGAAGACATC |
| G3-COL1A1_R2 | CAGATCACGTCATCGCACAAC |
| G4-MAGI1_F | GGTCGGATTATTGAGGGGAGC |
| G4-MAGI1_R | ATGTCAGAATGGGATTTGTTGGT |
| G5-SIX-2_F2 | CCTGCGAGCACCTTCACA |
| G5-SIX-2_R2 | CTCGATGTAGTGTGCCTTGAG |
